# T-cell epitope prediction and immune complex simulation using molecular dynamics: state of the art and persisting challenges

**DOI:** 10.1186/1745-7580-6-S2-S4

**Published:** 2010-11-03

**Authors:** Darren R Flower, Kanchan Phadwal, Isabel K Macdonald, Peter V Coveney, Matthew N Davies, Shunzhou Wan

**Affiliations:** 1 Life and Health Sciences, Aston University, Aston Triangle, Birmingham B4 7ET, UK; 2 Oxford Biomedical Research Centre, The John Radcliffe Hospital, Room 4503, Corridor 4b, Level 4, Oxford, OX 3 9DU, UK; 3 OncImmune Limited, Clinical Sciences Building, Nottingham City Hospital, Hucknall Rd. Nottingham, NG5 1PB, UK; 4 Centre for Computational Science, Chemistry Department, University College of London, 20 Gordon Street, WC1H 0AJ, London, UK; 5 SGDP, Institute of Psychiatry, King's College London, De Crespigny Park, London, SE5 8AF, UK

## Abstract

Atomistic Molecular Dynamics provides powerful and flexible tools for the prediction and analysis of molecular and macromolecular systems. Specifically, it provides a means by which we can measure theoretically that which cannot be measured experimentally: the dynamic time-evolution of complex systems comprising atoms and molecules. It is particularly suitable for the simulation and analysis of the otherwise inaccessible details of MHC-peptide interaction and, on a larger scale, the simulation of the immune synapse. Progress has been relatively tentative yet the emergence of truly high-performance computing and the development of coarse-grained simulation now offers us the hope of accurately predicting thermodynamic parameters and of simulating not merely a handful of proteins but larger, longer simulations comprising thousands of protein molecules and the cellular scale structures they form. We exemplify this within the context of immunoinformatics.

## Review

As Donald Knuth, the famous polymath of modern-day computer science, wrote in the forward to the book “A = B”: *Science is what we understand well enough to explain to a computer; Art is everything else we do.* Whatever its verity, this statement has a clear and compelling appeal. The human mind, which has evolved within the macroscopic world to understand macroscopic phenomena and predict macroscopic behaviour, cannot completely grasp nor truly possesses a complete intuitive understanding of the microscopic world of atoms and molecules; a place which exists at the intersection of two worlds: the quantum world and the world of conventional, large-scale physics. In venturing to gain a scientific understanding of these elusive, recondite worlds far beyond the limits of our direct experience, we seek to do synthetic reductionism: dissecting phenomena associated with the nature and behaviour of the biological molecules comprising the systems we study, and then building mathematical models capable of predicting the more complex behaviour of the systems emerging from these components.

It is only through accurate and robust prediction, that we can be sure that we have attained any degree of true understanding. The problem with bioscience in general and immunology in particular is that our knowledge of the basis of adaptive immunity is largely compiled from indirect sources. Such sources include *in vitro* experiments, which are performed in controlled yet often highly artificial conditions far removed from the biological context of the whole organism.

To a harsh eye and a harsh mind, the interpretation of cellular function within the immune system is particularly indirect and inferential, being largely based on the use of flow cytometric detection of surface markers or the cytotoxic or proliferative behaviour of a bulk population of cells. All such experiments ultimately give insight only in a most circuitous manner. To pass to the practical, there has been much recent biomedical interest expressed in computational tools for the analysis of epitope-mediated immunogenicity [1-4]. The adaptive immune system saves us from the death and disability engendered by infectious disease. The adaptive immune response functions to destroy invading pathogens. Effectively distinguishing foreign or non-self molecules from host or self molecules is vital. One half is the humoral immune response: antibodies, produced by B cells, bind to antigens on the surfaces of invading microbes. The cell-mediated immune response forms the other half of adaptive immunity; here activated T cells react against foreign antigen presented on the surface of other cells.

Extensive, and continuing, work has been undertaken to develop novel epitope prediction methods, based on a variety of reliable and robust computational methods. The main focus has been the quantitative prediction of peptide-MHC interactions, the necessary precursor to the recognition of epitopes by T cell receptors, and the identification of continuous and discontinuous B-cell epitopes [5-8]. Such approaches seek to combine the best aspects of experimental and informatic science.

Informatics, in the form of immunoinformatics, thus offers a considerable variety of tools and techniques for undertaking the rapid, robust, and accurate computational identification of epitopes. By using such *in silico* approaches, computer-based prediction methods can significantly increase the celerity of T-cell and B-cell epitope discovery, with a concomitant dividend for vaccine design and discovery. With an ever-increasing number of pathogen genomes now available, the mapping of B-cell and T-cell epitopes, both computational and experimental, is becoming a central issue in vaccine discovery [9-17].

 However, using epitope mapping and epitope prediction makes understanding the structure or function of a particular pathogen gene essentially irrelevant. Nonetheless, gaining insight into function can add value to the exercise, allowing evolutionary rationales to be posited, for example. Mapping or prediction works solely with the physical structure of the protein, either *in vivo*, *in vitro*, or *in silico*, and insights into the role played by a protein in its organism of origin or within the context of host-pathogen interactions are simply not needed.

By far the most successful prediction strategy has been the data-driven prediction of T-cell epitopes. In a pivotal retrospective analysis, Deavin* et al. *[18] compared several early direct T-cell epitope prediction methods, without finding a single method possessed of sufficient accuracy to be deemed useful. T-cell epitope prediction typically involves defining the peptide binding specificity of specific class I or class II MHC alleles and then predicting epitopes *in silico*. Today work focuses on predicting class I MHC-peptide binding affinity. At least for well-studied class I MHC alleles, such methods work well [19,20].

However, for prediction of all immune epitope data other than class I MHC peptide binding, results have been unsatisfactory and substandard. Over the last few years, several comparative studies have shown that the prediction of class II T-cell epitopes is usually poor [21-23]. Likewise, both structure- [24] and data-driven [25] prediction of antibody-mediated epitopes have again been shown to be poor. Moreover, irrespective of the poor reported predictivity, there are several other problems, albeit different for T-cell and B-cell epitope prediction.

A quite different approach to obtaining predictions of peptide:MHC binding is based on so-called molecular dynamic simulation. This technique attempts to calculate the free energy of binding for a given molecular system, which is closely related to experimentally observable quantities such as equilibrium constants or IC_50_s. It has the advantage that, in principle, there is no reliance on known binding data, as it attempts the *de novo* prediction of all relevant parameters given certain knowledge of the system. Essentially, all that is required is the experimentally determined structure, or a convincing homology model, of a MHC peptide complex.

Explicit solvent Molecular Dynamics (or MD) is an atomistic means of simulating the behaviour at room temperature of one or more solute molecules, such as an MHC protein, of defined geometry surrounded by an environment of solvent and ions over a timescale of one to several thousand nanoseconds. As we shall see below, for a system evolving over time, MD provides an unsurpassed and unsurpassable level of detail for all dynamic behaviour.

## What is molecular dynamics?

To answer this question fully is difficult, yet we can seek to adumbrate at least a partial answer below. MD describes in detail the individual and collective motion of atoms within a molecular system [26]; thus MD can provide a dynamic, rather than a static, picture of biomolecular systems. In the context of affinity and epitope prediction, MD has the advantage that it is not data-driven, as it attempts the *de novo* prediction of all relevant parameters given knowledge the system’s starting structure, be that an experimental structure or a convincing homology model of ligand receptor complex. Unlike other methods, MD can, in principle at least, account for both explicit solvation and the intrinsic flexibility of both receptor and ligand.

For large molecular systems comprising thousands of atoms, many of the more sophisticated modelling techniques, which often describe the potential energy surface in terms of quantum mechanics, are too demanding of computer resources to be useful. The Born-Oppenheimer approximation states that the Schrodinger Equation for a molecule can be separated into a part describing the motions of the electrons and a part describing the motions of the nuclei and that these two motions can be studied independently. MD is able to compute the equilibrium position of a classical multiple body system. It is assumed that the atoms of the system are constrained by an inter-atomic potential energy force field. Each atom in the simulation is treated as a point mass and Newton's equations are integrated to compute the motion of the N atoms comprising the system. Thus:

m_i_ is the mass of atom i, r_i_ is the Cartesian 3-vector of atom i, the second derivative of r_i_ is the acceleration of atom i, and **F***_i_* is the force vector acting on atom i. We need only define the initial configuration of the system, defining an initial set of atomic coordinates and setting the initial atom velocities at random. At regular time intervals thereafter, we are able to resolve the equation of motion as represented by equations for each atom. The forces on the atoms are calculated from the gradient of the potential energy function. The simulation progresses as new positions and velocities are computed, and the atomic coordinates updated.

Several algorithms exist for integrating the classical equations of motion for these prodigious point swarms; and include the Verlet algorithm, the velocity-Verlet, and the leap frog algorithm. The time step chosen for the integration should be considerably less than the fastest event simulated. About the fastest event is the vibration of a bond to hydrogen, which is of the order of 10 femtoseconds (fs); for comparison, a significant conformational change or rearrangement will occur on a time scale in the microsecond range. Thus hydrogen vibration sets an implicit scale for a minimum time-step. When bonds involving hydrogen are not constrained in simulations, the characteristic time-step will be 1fs. When bonds to hydrogen are constrained, or the hydrogens themselves are not simulated explicitly, as is the case for a so-called united atom model where hydrogen atoms and their bonded heavy atom are treated as a single interaction centre, then the time step can increase to the order of 2-5fs.

To measure an observable quantity we must be able to express this as the position in a phase space of dimension 6*N. The information within our system is largely contained within the potential energy function, also known as a so-called force field, which takes the form of a simple penalty function for most atomistic biomolecular simulations. Molecules are treated as pseudo-mechanical assemblies comprised of many simple elements: atoms are modelled as spheres, bonds modelled as rods, and bond and torsion angles modelled as flexible joints. Thus force fields consist of harmonic springs for bonds and bond angles, supplemented by torsion profiles for dihedral angles. Atoms are modelled as spheres, typically with constant point charges localized at the atomic centres. Terms which describe various types of interaction between atoms, including Van der Waals and electrostatic, and possibly hydrogen bonding, are also part of such force fields.

There are many extant force fields; biomolecular force fields are intentionally parameterized to be multipurpose. The most popular force fields include MM3 [27] for small molecules, and GROMOS [28], AMBER [29-31], and CHARMM [32,33] for proteins. Current force fields remain imperfect, and developing more physically accurate multipurpose polarization force fields is challenging. There are doubtless many cryptic effects that are not dealt with appropriately, such as salt or pH effects; there is therefore a continuing effort to improve the description of interatomic and intermolecular forces. Mono-valent ions and solvent interactions are probably described adequately. The backbone description would benefit from geometry-dependent polarization and electrostatic terms. Divalent ions are currently problematic for most force fields as their first ligand shell is highly activated through polarization and charge-transfer; all such complex phenomena are neglected by standard force fields. One means of addressing this confounding issue is to combine quantum mechanical effects into the MD simulation. We shall navigate a brief discussion of this subject below.

As a technique, MD has for some time found use in the prediction of the affinity or binding free energy (ΔG_bind_) of macromolecular-ligand interactions [34]. Generally though, such endeavour has been instigated and justified as demonstration of the potential of MD rather than as routine tool delivering prospective rather than retrospective predictions. There are many reasons for this. One is the scepticism of experimentalists, reluctant as they are to surrender their hegemony to the computer. Another reason is the practicalities of routine simulation on a scale large enough to be useful. Until relatively recently, the computing resources necessary to sustain simulations of sufficient size and duration to obtain affinities has greatly restricted the general exploitation of such techniques. In spite of this there has been much interest in pursuing such a strategy, as MD has the advantage that known binding data is unnecessary, since the approach seeks to calculate all relevant parameters. For a simulation of a macromolecule-ligand complex, all that is required is an initial experimental protein structure, or a reliable homology model.

**Figure 1:**
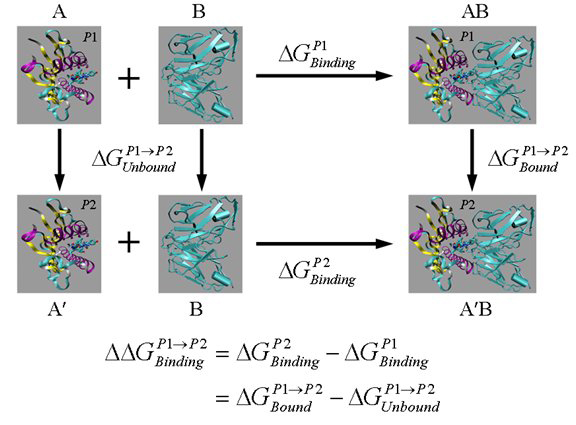
**A thermodynamic cycle for free energy calculation.** AB, A′B and A, A′ are the bound and unbound states of two different, but similar molecules (shown here as peptides p1 and p2 complexed with MHC) binding with the same molecule B (shown as TCR). Here  and  are the binding free energies of A and A′ to B, while  and  are the free energy differences between the two molecules in their bound and unbound states, respectively. The vertical legs above are ‘alchemical’ paths which transform one molecule into another, while the horizontal legs are ‘chemical’ paths which describe the binding process of one molecule to another.

The definition of free energy (G), as derived from statistical mechanics, is framed in terms of the partition function. This theoretical definition is however of little practical value for most types of calculation. What one can calculate more easily, however, is the free energy difference ΔG_A->A’_ between two states A and A’. In the vertical legs of Figure 1, states A and A’ are shown as the peptides p1 and p2 complexed with an MHC in the unbound state, and as TCR-p1-MHC and TCR-p2-MHC complexes in the bound state. Methods exist able to evaluate these energy differences, each predicated on a different set of assumptions and approximations [35]. The semi-rigorous prediction of ΔG_A->A’_ can be approached using free-energy-perturbation (FEP) calculations; or it can be undertaken using thermodynamic integration (TI). See Figure 1. The binding free energy difference ΔΔG_binding_ can be calculated, which is more important as it shows the relative affinities of two similar molecules A (p1-MHC) and A' (p2-MHC) binding to the same molecule (TCR). These compute-intensive approaches use the ensemble average of an energy function, which is itself a function of the coordinates in configuration space.

Another strategy uses the horizontal legs in Figure 1 to calculate the binding free energy ΔG_bind_. Individual free energies for the complex, the isolated ligand, and the isolated receptor are calculated separately from MD trajectories. The overall binding free energy is given by:

ΔG_bind_ can be reduced to a set of contributions, each defined so that cross terms can be neglected. Energetic contributions are typically derived from a limited set of structures rather than from extensive ensemble averages. Entropic contributions arise from lost intra-molecular flexibility and molecular motion. A continuum solvent model solved using a linear Poisson-Boltzmann equation replaces explicit electrostatic contributions made by water. Contributions made by non-polar atoms to the desolvation energy are also calculated empirically.

A widely used example of such an approximate technique is the so-called MM/GBSA method [36,37], which combines energies calculated using molecular mechanics with implicit solvation models. Averaged properties are calculated from a sampled MD trajectory, with entropic contributions obtained using normal mode analysis. Receptor-ligand complexes with significant structural variation can be studied using this approach.

where *G_molecule_* is the free energy of the complex, or the isolated receptor, or isolated ligand; *E_MM_* is the energy calculated from the MD trajectory using an empirical force field; *G_sol_* is the solvation energy; and -*TS* is the entropic contribution. Angle brackets denote an average over time, or another continuous parameter, in this case an MD trajectory. When calculating the binding free energy difference, it may be assumed that the standard entropy change is similar for both models and can be neglected. Other popular approaches to structural simulation include the use of normal mode analysis (NMA) [38], which obtains the simple harmonic motions of a protein about local energy minima, or MC sampling [39].

## Molecular dynamics simulations of the major histocompatibility complex system

A considerable time has now passed since the first MD simulations on an MHC-peptide complex were undertaken. During this time the availability if not the ease of use of dynamic simulation has increased out of all recognition [40]. Compared to some obscure proteins, much work has been expended on simulating the Major Histocompatibility Complex and its interaction with peptides and the T-cell receptor; yet, when compared to other, more famous proteins, relatively little work seems to have been done. Researchers, experimentalists or theoreticians, are often loathed to abandon any system which works for them.

Delisi and co-workers, were the first, or at least among the very first, to make use of MD as a tool to explore peptide-MHC binding. Subsequently, they developed several different methods [41-52]. They partly focussed on accurate docking using MD and partly on determining corresponding free energies. Didier Rognan also made important early contributions in this area [53-58]. He found that the simulated dynamic properties of the MHC-peptide complexes, such as solvent accessible surface areas and hydrogen bonding patterns correlated well with binding data. He could discriminate effectively between tightly anchored binding peptides and weaker non-binders.

More recently, several workers have begun using MD methodology to analyse the characteristic features of peptide-MHC complexes and to predict the dynamic behaviour and thermodynamic stability of the system. These methods have been used to evaluate Class I [59,60] as well as Class II [61]. Painter *et al*. [62], following on from a study by Zacharias and Springer [63], compared the peptide bound and non bound state of the MHC molecule using molecular dynamic simulation technology.

Yaneva et al. performed MD simulation studies of HLA-DR3 with and without invariant chain-associated peptide (CLIP); finding that larger conformational changes of alpha-helices flanking the MHC binding groove occur when CLIP is not present [64]. Additionally, others have used the MHC as a suitably sized test bed for developing novel aspects of MD methodology, including both simulation methodology [65] and solvation [66]. In such work, the MHC-peptide complex provides a convenient example of a binary molecular complex. By combining MD simulation, peptide/MHC binding assays, and T-cell activation, Knapp and co-workers demonstrated that the N-terminal peptide flanking region of class II MHC binding peptides significantly enhances and modulates peptide immunogenicity [67].

Michielin and Karplus [68,69] undertook a study that made use of a whole array of *a posteriori* or *post hoc* approximations to compute relative free energy differences between bound TCR-pMHC complexes with peptides mutants. This approach used so-called thermodynamic integration to calculate the free energy. Despite the stated accuracy in its prediction, in many ways this study was most unsatisfying, laden as it was with unjustified methodological approximations: approximations which could only have been applied if the result was already known.

The study of Cuendet and Michielin [70] is a follow up to this investigation. The characteristic half-life of a TCR-pMHC complex can last several seconds: a time far beyond the traditional range of atomistic simulation. Their study afforded a new, if incomplete, insight into the mechanism underlying disruption of the TCR-pMHC complex, using a steered molecular dynamics (SMD) protocol [71]. This is an example of non-equilibrium dynamics, where the motion along the reaction coordinate is progressively driven by an external potential. Such approaches include both the early targeted MD studies of  [72-74] and full SMD [75-82].

**Figure 2:**
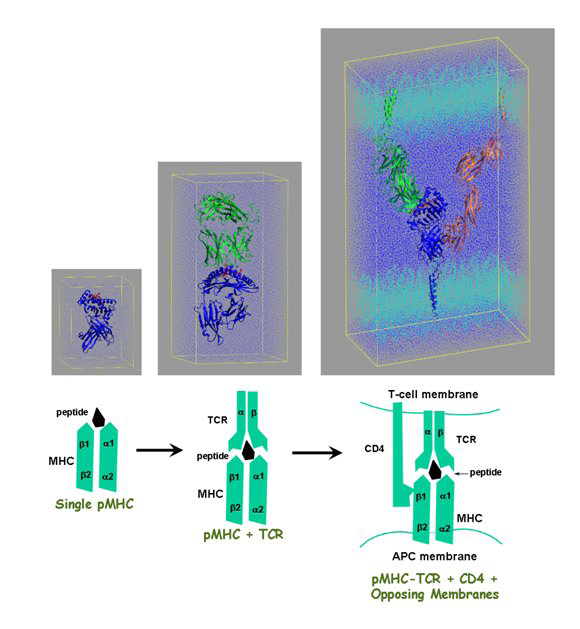
**the escalating scale necessary to simulate large-scale immunological phenomenon.** (A) Model of the MHC alone, equivalent in size to the simulation undertaken in [150]. (B) Model of the MHC complexed to the TCR, equivalent in size to the simulations undertaken in [151]. (C) Complete model of a unit of the immune synapse, comprising MHC, TCR, peptide, CD4, and two opposing sections of membrane, as simulated in [141].

Our own work, spearheaded by Shunzhou Wan, represents efforts to address the potential of large scale MD, allowing us to evaluate a series of increasingly complex molecular systems [83-87]. Our simulations escalated from an isolated MHC molecule [83], via the TCR-pMHC complex [84,85], where the computed structural properties and thermodynamic binding parameters are in good agreement with experiment, through to simulating a recognition complex comprising pMHC, TCR, and a CD4 molecule embedded in two opposing patches of membranes [87]. See Figure 2.

Since this TCR-pMHC-CD4 complex is the smallest viable complex able to trigger transient calcium signalling [88-91], this last undertaking is the first stage in reaching our intermediate goal of simulating a full immune synapse (IS): the adhesive junction formed between T cells and professional antigen-presenting cells (APCs). The immune synapse plays a crucial role in mediating initial immune recognition relaying information from APC to T cell. This junction is a highly organized, spatio-temporal arrangement of receptors and accessory molecules of many types. At least four key kinds of molecules are involved in mediating the formation of the mature synapse [89,92]: lymphocyte function-associated antigen-1, intercellular adhesion molecule-1, TCR, and the pMHC. The IS controls the strength and duration of signals that can activate T cells, and enhances the accumulation of peptide-major histocompatibility complexes (pMHC) at the interface, thereby enhancing engagement with TCR and subsequent cell signalling.

Experiments [92] have revealed the time frame in which the IS forms, and the shape of the mature immune synapse. The physical phenomenon has also been modelled by reaction-diffusion equations and by Monte Carlo (MC) simulation using statistical-mechanical models [93-95]. However, little is known about the spatial arrangement of individual molecules at the IS [89,92], and how receptor clustering is orchestrated. A complete view of the spatio-temporal dynamics of the IS remains beyond current experimental methods. Multi-scale molecular dynamic simulation may be the most tractable way to gain a complete understanding of this complex supra-macromolecular event.

This is the first step in a long journey which will hopefully lead us to a full dynamic model of the immune synapse. The immune synapse involves tens of thousands of molecules not tens of thousands of atoms. The timescale for its formation and maturation is about 30 minutes [92]. We have taken the next step by simulating an extended model, in which 4 sets of TCR-pMHC-CD4 complexes are included. This fully-atomistic model, which includes explicit water and lipid molecules, is approximately one million atoms in size. We have undertaken 10 nanosecond MD simulations of the system and the analysis is in progress. Mechanistic insights relating to component interactions are gained from such simulations. With further information on the protein-protein and protein-membrane interactions, extrapolation can be made to more and more realistic IS models. Many insights can be gained from these models about the dynamics of receptor binding and trafficking and how these influence signal transduction by endogenous and exogenous ligands.

## Comparison of molecular dynamics to experiment

Generally speaking, MD simulations face two significant limitations. First, the inadequate sampling of conformational space which reflects the short time scale of MD simulations compared with those exhibited by biological processes. This is being addressed by the continued emergence of widely deployed supercomputing and widely accessible parallel MD code. Second, despite increasingly sophisticated parametrization, most, if not all, empirical force fields remain highly approximate [96-98]. We are thus still frustrated by concerns over the intrinsic validity of such simulation exercises. Many approaches have been tried to circumvent these problems, but only with limited success, since almost any attempt to reach longer time scales will result in more and more approximations.

Previous attempts to utilise MD to investigate peptide-MHC interactions have foundered on technical limitations. There is a tension between what we would wish for and what is available to us. A race exists by which the bigger the computing device we have, the bigger and longer the simulation we wish to run. Since the scale of biology is vastly greater than the largest realizable simulation, it is a race which simulation always loses. Our ambition invariably exceeds all possible achievement.

Many approaches have been tried to circumvent these problems, but only with limited success, since almost any attempt to reach longer time scales will result in more approximations in the model. While many methods link thermodynamic properties to simulations, they take an unrealistically long time. Moreover, there is a need for long simulations able to accommodate both slow and very fast – protein conformational change and enzymatic transition states - motions seen in biological simulations. Widely available serial computing has allowed the MD simulation of biologically interesting systems of moderate size. A basic free energy simulation requires at the very least ten nanoseconds of simulation time. This would take hundreds of hours per nanosecond of compute time on a desktop workstation. A simulation of, say, a score of peptides might occupy a whole machine for many years.

Thus there is a clear imbalance between generally-available computing resources and the size of the task to be evaluated. One way to address such an imbalance is to use as large and as powerful a computer as one possibly can. This necessitates the use of tightly-coupled supercomputing to massively increase the computing resources available, as distributed computing of the screensaver type is simply not appropriate to MD problems.

Distributed computing, most famously epitomised by folding@home [http://folding.stanford.edu/], splits computing jobs in separate, autonomous tasks and distributes them across thousands or tens-of-thousands of individual computing nodes, most usually unattended personal computers left running out-of-hours. Folding@home and seti@home [http://setiathome.berkeley.edu/] are amongst the largest distributed computing efforts. Several kinds of computing study are readily addressable using such an approach, including certain biomolecular simulations. For example, a 20,000 atom simulation of the Villin Headpiece using 200,000 CPU's distributed around the world was run for a total of 500,000 nanoseconds in order to model the folding of the protein [99]. Because the simulation was probing the kinetic rather than thermodynamic properties of the system, the use of many different, independent, short-term trajectories run using CPU's without real-time communication was wholly appropriate. An MD simulation could not be run this way, only many copies of the same simulation.

However, a series of competing but ultimately complimentary technical advances has opened a potential new if not yet properly realised era of accessible and widely-deployed supercomputing. Supercomputing offers the opportunity to circumvent logistic limitations by allowing high performance, massively parallel implementations of MD codes to run on large integrated computing systems with 512 or 1024 nodes.

However, the ready availability of dedicated, truly large-scale supercomputing running from national centres is not what once was hoped. Several other alternatives are, however, becoming more widely accessible. These alternatives are partly composed of new computing platforms and partly of novel, seamless means of deploying supercomputing capacity.

In the vanguard of such approaches has been the advent about a decade ago of so-called GRID computing. This refers to an ambitious if never fully realised effort to develop an environment in which individual users can access computers, databases and even experimental facilities simply and transparently, without having to consider the location or identity of such facilities. The GRID has partly given way to the cloud computing. Cloud computing is a term for the delivery of modern-day internet computing, yet maintains much commonality with a more traditional client-server paradigm. Typical cloud computing provides applications accessed via web browsers, with the underlying software and databases housed on servers. Cloud is here used in an abstract and metaphoric sense, and derives from the use of cloud drawings to represent the underlying structure of complex networks, such as the telephone system and the internet. Importantly, for comparison with GRID computing, details are supposedly transparent with respect to users; users who are thus freed from the need to understand, let alone gain expertise in, the underlying technological infrastructure. This is a natural extension of the specialisation of Adam Smith’s pin factory; knowledge becomes ever more specialised, to the point where we need know only what we need to know, and all else is done for us. Though why some disciplines gain a mystique - medicine or IT for example - while others - such as car maintenance or scientific research - do not, is harder to understand. Key is that cloud computing provides resources via the Internet which are dynamically scalable and often virtualized.

Another important development is the GPU. A GPU or graphics processing unit contains large numbers of arithmetic units able, amongst other things, to accelerate greatly numerically intense MD simulation. It seems ironic that science, from which emerged the business of computing, has now become the poor relation to a branch, albeit a lucrative branch, of the entertainment industry. The coupling of modern GPU hardware and GPU-specific programming languages has made exploiting this technology relatively facile, and thus its deployment has burgeoned in the last few years. GPUs can support efficient data-parallel algorithms that scale to hundreds of tightly coupled processing units; an ideal situation given the requirements of so much of MD simulation. For example, GPUs greatly facilitate the evaluation of non-bonded interactions in MD simulations, allowing performance over twenty times that of an isolated CPU.

To a greater or lesser degree, software forms an important part of almost all scientific research projects, yet most specialist research software is only just fit for purpose, at least when it is compared to equivalent programs available for the worlds of business. Much is made of open-source software, and much is highly innovative, but it is not developed to the high standards expected of commercial software. Code is often hard to understand and even harder to maintain and develop, with inconsistent levels of documentation and little orchestrated version control. Software designed to do science, as opposed to say Microsoft products whose use in science is secondary and incidental, is continuously in the process of being either rewritten or written again from scratch, as their programmers move onto new employment on the ridiculously short three-year post-doc turn around. This is a staggering waste of the already grossly inadequate resources put into science in general, and code-development in particular. MD code suffers less than many areas in this regard, yet does not escape the problem entirely, particularly when coming to terms with parallelisation and the need to rewrite code so that it can capitalise on potential improvements in speed and efficiency. Increasingly, software, such as NAMD [100-102], is specifically developed to run well under parallelisation, making use of the enormous innate synergy in efficient scaling. Older programs, such as native CHARMM or AMBER, scale poorly and porting them to a parallel environment has often proved problematic.

Thus, computing power increases, and through such innovation, will continue to increase in some sense for the foreseeable future, yet this does not solve all problems, and many remain. However much computing we have, we can always envisage simulations of a greater size or duration and quickly exhaust whatever resource we have available. Likewise, while running larger simulations for longer does much to mitigate the effects of inaccuracies of method, they cannot compensate completely.

A force field’s performance relies on the mutual cancelling of errors. This means that an energy penalty function must be balanced in the system under study and the parametrization must be accurate. If errors do cancel, then the correct global minimum of the system will be found: not all details are necessarily correct, but the overall description is meaningful. The longer and the larger the simulation, or the more qualitative the answer sought, the more likely that such a situation will be achieved. If errors do not cancel, a global minimum will prove elusive; as a result the system will rapidly degrade [103]. Clearly, longer and larger simulations, such as those offered by supercomputers and similar methodology, as described above, offer an equal benefit in terms of parameterisation. As timescales and the total size of simulations increase, the more likely we are to get cancellation of errors. In essence, we will observe a general, smoothing phenomenon: isolated temporary but gross local errors, in space and time, will largely disappear under the mass of more accurate observations from the majority of the simulation.

Obviously, and bearing in mind what we have said above, some well-judged sagacity is required to use the results of MD simulations properly. If an accurate experimental starting structure is available, MD can often improve molecular interactions and local backbone and side-chain conformations. However, inadequacies inherent with biomolecular force fields as well as deficiencies in available sampling protocols mean that MD cannot predict protein structures without experimental input. Local conformational traps associated with standard MD can be overcome by enhanced sampling techniques, such as locally enhanced sampling, replica exchange, or targeted MD [81,82]. Robust sampling is also critical to obtain reliable results from free energy calculations [104]. Unrealistic initial models will typically become deformed by MD. If a structure has an incorrect geometry confined by large energy barriers, MD will not move it from its initial geometry.

Moreover, the MD trajectories that result from any simulation should be compared rigorously with available experimental data in order to assess both the accuracy of the simulation and the implications of any structural or calculated energetic change. Historically, the results of simulations have always been compared to crystal data. In this context, the analysis of a restricted subset of atoms or simple root-mean-square distances may mask problems. However, increasingly the results of larger and more sophisticated simulations must be compared to other experimental data: typically either thermodynamic quantities, such as affinities, or time-dependent quantities, such as diffusion coefficients.

Although the immune system is hierarchical, and exhibits much emergent behaviour, at its heart are straightforward molecular recognition events that are indistinguishable from other types of biomacromolecular interaction, such as those between antagonist and receptor or enzyme and inhibitor. Epitope binding to an MHC is, in terms of underlying physico-chemical phenomena, identical to binding of small molecule drugs to protein receptors. Isothermal titration calorimetry (ITC) is a powerful technique to study protein-ligand interactions: it is a method which measures stoichiometry directly, making interpretation relatively and reasonably facile [105-107]. Moreover, labelling, chemical modification, immobilization, and competing molecules are not required, making it both sensitive and rapid.

During binding, heat will be produced or absorbed. ITC measures these heat changes directly, giving a complete picture of a reaction’s thermodynamic parameters, allowing the accurate determination of equilibrium dissociation constants (Kd), stoichiometry (n), enthalpy (∆H), entropy (ΔS), and heat capacities (ΔC_P_). Thus ITC goes beyond simply providing binding affinities, in the form of equilibrium dissociation constants as other techniques also do, and can help elucidate the underlying molecular mechanisms of binding interaction. Such insight is currently unobtainable using other binding assays, such as surface plasmon resonance (SPR). ITC is, at least for the majority of commonly encountered biochemical reactions, quickly becoming the only real choice for characterizing biomolecular interactions. It is the only technique to measure binding enthalpy directly, thus obviating the necessity of van't Hoff analysis.

Obviously, since no technique is perfect, ITC will of course impose its own restrictions, limitations, and boundaries. The method can measure affinities but only within a characteristic if wide range: 10^-2^ to 10^-9^ M; ITC needs to make use of competitive binding, i.e. through the displacement of weaker ligands, in order to measure affinities below the nanomolar: 10^-9^ to 10^-12^ M. However, this highly automated technique doesn’t impose unacceptable limitations on molecular weight, or the choice of buffer, and can cope easily with turbid or coloured solutions, including particulate ones.

**Figure 3:**
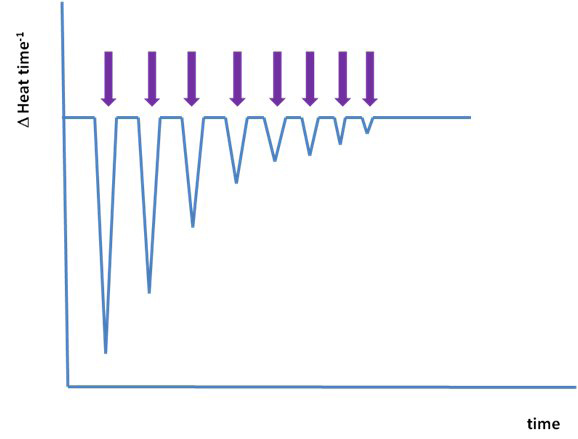
**Schematic of an ITC experiment.** Evolution of an ITC experiment; each arrow indicates a separate injection of ligand.

Typically, a solution containing the ligand, such as a peptide, is titrated into a solution of its receptor, for example an MHC protein. The heat produced is monitored over time. See Figure 3. Each peak is a heat change when a small volume of the ligand is injected into the ITC reaction cell. As the titrant increases, the heat change produced should be directly proportional to the extent of binding. As the system becomes saturated, heat changes dwindle, leaving only heats of dilution. The binding curve can be deduced from this plot yielding values for 1/K_d_, n, and ΔH.

Our experience of ITC, though limited, indicates that MHC proteins are indeed amenable to study by ITC. We have looked at two systems – HLA-A*0201 and HLA-C*0701 – and we observed significant heats of binding. However, for reasons that have yet to yield to an obvious rational explanation, the overall set of experiments did not allow us to calculate dissociation constants, nor entropies and enthalpies. Possibilities for this abounded. The most compelling was that the system may not have reached saturation, and that this may result in part from a much more complex binding phenomenon than has been supposed previously. Nonetheless, our results are consistent with the assertion that ITC can generate a much more complete decomposition of the peptide-MHC system than has been available previously. This provides a description which will be much more useful for all types of prediction, but most especially for MD simulations and the richness of its output. We are, given the opportunity, largely in place to predict and measure the full range of thermodynamic features of binding for the MHC-peptide system; only time and resource stands between us and that tantalising prospect.

## Competing technologies: virtual screening, quantum mechanics, and QM/MM simulations

Several other, similar computational techniques have been applied to the MHC-peptide system. In the next few paragraphs, we shall briefly survey some of the more interesting and some of the more relevant. These approaches form part of a spectrum of methods varying enormously in complexity from simple molecular descriptors, through empirical scoring functions, Potentials of Mean Force, Force Field or Molecular Mechanics Methods, MD and size-limited Quantum Mechanics, and ultimately onto full scale QM/MM and Free Energy perturbations.

In this transition from atom counts to full MD, there is a remarkable amplification of required computing. What we seek is some pragmatic solution to the accuracy gained vs time taken equation. The point at which one stops on this spectrum is contingent upon the system being evaluated, the number of peptides being evaluated, and the logistics of available computing power.

The term Virtual Screening (VS) derives from pharmaceutical research, where it is used as an alternative to high throughput screening: it uses predicted ligand-receptor interactions to rank and/or filter molecules [108]. Structure-based Virtual Screening is, in essence, the repeated static docking - and subsequent empirical scoring - of sets of small molecule structures to a biomacromolecular target. These sets can be substantial, reaching the dimensions of databases. Some see VS as simply verifying that molecules match the dimensions of the binding site. Others doubt all attempts to use structure to enlighten the discovery of ligands. While arguments regarding flexibility and large-scale rearrangements seem compelling, they merely act as a challenge to the discipline. Virtual Screening is a technique with wide applicability and a burgeoning track-record of success, at least in area of identifying small molecule ligands of macromolecular targets, yet should also help prosecute the identification of epitopes.

Speaking generally, and when viewed as a chronological procedure, Virtual Screening can be separated into five phases, beginning with the x-ray structure or homology model of a target protein. This is combined with potential agonists, antagonists, or inhibitors, or, in our case, potential peptide epitopes. These should have been carefully pre-screened for undesirable structural characteristics inconsistent with those of known drugs. The resulting set of ligands is then docked into a binding site model and scored for some appropriate correlate of binding. This process should discriminate between a small group of ligands with appreciable binding affinity and the bulk of ligands which will lack the ability to bind. The top handful of these ranked hits are selected, bought or synthesized, and assayed experimentally. Likewise, the more sophisticated, and thus, generally, time consuming, are our methods for evaluating the scoring phase of the virtual screening process, the more likely we are to screen accurately. If we want to dock a few tens of structures, then we can afford to expend a great deal of time on this process, but if our goal is to dock the peptide fragments representing a whole genome, then the practical limitations of computer time will reduce this to a minimum.

Several workers have sought to apply VS to MHC-peptide binding. Docking performs well when distinguishing potent ligands from non-ligands lacking all affinity. However, it does poorly when predicting binding affinity; particularly so for MHC binding prediction.

FRESNO is a now-ageing virtual screening methodology developed by Didier Rognan. It relies on a simple physicochemical model of peptide-MHC interaction [109,110]. This model was trained using binding data and experimentally-derived 3D structures for the MHC alleles HLA-A0201 and H-2Kk. The inability to predict binding constants directly from simulations, at least in a short time, has led some to combine calculation with statistics. Regression-based scoring is predicated on the assumption that the total free-energy is approximated by the sum of several separately-calculable contributions. Usually, regression coefficients are determined by MLR, PLS, or some AI approach. An initial training set of receptor-ligand crystal complexes and corresponding experimental binding affinities is used to train the tunable potential energy function. As with all data driven approaches, the contingent accuracy and the transferability to new congeneric series depends on the nature of the training set.

Rognan and coworkers found that lipophilic interactions contributed the most to HLA-A0201-peptide interactions, whereas H-bonding predominated in H-2Kk recognition. Cross-validated models were afterward used to predict the binding affinity of a test set of 26 peptides to HLA-A0204 (an allele closely related to HLA-A0201) and of a series of 16 peptides to H-2Kk. They concluded that their tuned scoring function could predict binding free energies using these. More recently, Rognan and colleagues found that, for predicting the binding affinity of 26 peptides to the Class I MHC molecule HLA-B*2705, FRESNO out performed six other, now rather out-dated, methods. The success of FRESNO suggests that optimising screening functions rather than using totally generic versions may prove a successful route, at least within a chemical area or protein family. This chimes with our experience using virtual screening approaches for MHC peptide binding prediction.

Turning now to bioinformatic based approaches, others are using amino acid pair potentials, initially developed to predict the fold of a protein, to identify those peptides which will bind well to a MHC. Margalit and Colleagues have proposed a number of virtual screening methodologies [111,112], each of increasing complexity. They used amino acid pair potentials, originally developed by Miyazawa and Jernigan [113], to evaluate the inter-protein contact complementarity between peptide sequences and MHC binding site residues. They presented an analysis of peptide binding to four MHC alleles (HLA-A2, HLA-A68, HLA-B27 and H-2Kb), and were successful in predicting peptide binding to MHC molecules with hydrophobic binding pockets but not when MHC molecules with charged or hydrophilic pockets were investigated. Again focussing on Class I alleles, a more recent study from this group [114] used an updated set of statistical pairwise potentials.

More recently, Davies and co-workers looked at a large dataset of MHC-structures using static energetic analysis following energy minimization, partitioning interactions within the groove into van der Waals, electrostatic and total non-bonded energy contributions [115]. They found that the whole peptide defined specificity, highlighting the lack of dominant interactions between peptide and MHC as there was no statistical separation in the performance of a set of disjoint, yet statistically-degenerate, models.

Class II has also been investigated using Virtual Screening methods. For example, Swain developed a threading method and applied it to Class II MHC peptide interactions. More recently, Tong and co-workers have used a virtual screening approach to address peptide binding to the MHC allele HLA-Cw*0401 [116]. They concluded that class II anchor residues were not restrictive, implying that binding pockets can house many more peptide sequences than is supposed solely from the prediction of anchor residues.

Within this context, two recent comparative papers have analysed the limitations implicit within attempts to predict this phenomena. Knapp *et al*. [117] combined different tools for side chain substitution, energy minimization, and interface scoring in an evaluation of class I peptide-MHC binding. For a binary distinction between non-binder and binder, their results varied considerably with the protocol they used, with outcomes ranging from near random, to accuracy of about 75%. Zhang, Peters, and co-workers [118], assessed several methods for class II MHC-peptide prediction showing them to be better than random, but still markedly inferior to best-in-class sequence-based prediction. They conclude that their results are consistent with the idea that only the use of measured binding data can yield satisfactory results.

Sadiq *et al*. reported recently a Virtual Screening approach that combines the core protocol behind VS but with significant technical enhancements that bring the full power of MD to bear on the problem [119]. They developed a highly automated workflow tool, their Binding Affinity Calculator (BAC), for free energy ranking, which enables them to automate and thus accelerate the process of docking and performing binding affinity prediction. Their system uses fully atomistic molecular dynamic simulations together with the well-understood molecular mechanics Poisson−Boltzmann solvent accessible surface area (MM/PBSA) free energy methodology, enabling them to calculate the free energy of binding of protein-ligand complexes.

Compared to empirical, molecular mechanics-type potential energy functions, Quantum mechanics (abbreviated universally as QM) provides a much more accurate physical description of atomic and molecular interaction, which is, at the *ab initio* level at least, substantially free of empirical parameterisation. It is well-known that although it can offer systematic improvements in quality by modulating atomic orbital basis sets and including electronic correlation effects, *Ab inito* QM is still effectively limited to gas-phase simulation of systems comprising no more than 30 to 50 atoms. Semi-empirical QM, long used to simulate organic small molecules, can, on the other hand, cope with much larger and more complex molecular systems. Examples include MNDO [120], AM1 [121,122], and PM3 [123,124].

However, it is not straightforward to extend QM calculations to biomolecules, whose conformational space arises from a complex spatial architecture of mutually-compensating intra- and inter-molecular interactions. Many biomacromolecules, including proteins and nucleic acids, would experience a very robust electrostatic environment without the mitigating effects of solvent screening. Unfortunately, the accurate modelling of the dynamic behaviour of solvent lies outside the orbit of current QM techniques. Here, the issue is not so much the intrinsic performance of the QM methods themselves, but deficiencies in the system being modelled.

Until recently, Semi-empirical QM methods have been little used to study proteins, mainly due to issues over reproducing the geometries of large systems and the heavy computational demands involved. From a QM perspective, and despite their great size, two factors make their simulation tractable: one is the lack of exotic chemistry, such as are evinced by organo-metallic compounds or transition elements, and the repetitive nature of its polymeric structure, and thus their concomitant intramolecular interactions, which allows for some simplification of calculation. Obviously, proteins can contain complexed metal ions but a large proportion do not, and with the exception of proteins containing metalloporphyrin heme and chlorophyll, many of the subtle electronic effects encountered with transition metals are absent.

Many attempts have sought to decrease the computational burden of using semi-empirical QM to model proteins: for example, the divide-and-conquer method [125], which, as its name suggests, partitions a protein into components, a technique much applied to biochemical systems [126-128]. Stewart reports the application of the sophisticated semi-empirical QM method PM6 to the simulation of several protein systems [129]. Here, self-consistent field (SCF) equations were solved using the so-called MOZYME method [130] rather than more computationally demanding algorithms.

QM/MM, which stands for quantum mechanics/molecular mechanics, is a hybrid approach supposedly combining the accuracy of quantum mechanical calculations with the speed of molecular mechanics. This allows the study of very large, physically-realistic systems inaccessible to straightforward QM with a rigour inaccessible to MM. Classical MD calculations are of the *O*(N^2^) while ab-initio Quantum Mechanics calculations usually scale *O*(N^3^) or worse. Another hybrid approach models the “interesting” parts of a protein system using QM, and the rest using MM. In our terms, the interesting region of the system would correspond to the peptide binding site of the MHC, and the rest would be the b2-microglobulin and a3 domains. Despite being compromised by issues of defining, both accurately and tractably, the boundary between the MM and QM regions of the system being modelled, QM/MM, has nonetheless generated much interest [131]. Thus far only there has only been one report of the application of QM/MM techniques to the MHC system [132].

## Towards useful and interesting molecular dynamics simulations

Powered and empowered by the availability of supercomputing, cutting-edge MD simulations can now tackle very large systems [133]. Most simulations of immunological systems have by contrast been small and meagre efforts. Even the largest such simulation is small even when compared to the state-of-the-art in biomolecular simulations, which in themselves are very small when compared say to the size of a cell or a sub-cellular organelle.

However, assisted by rapid advances in experimental imaging and quantitative proteomics, information can now be garnered from simulations that are beginning to approach the mesoscale rather than the scale of atoms [134]; we no longer need to restrict ourselves to the dry and uninteresting pico-second simulations of individual proteins that were the meat and drink of yester year, but can instead look to simulate in a reasonably realistic way supramolecular systems and even biologically meaningful cellular components.

In a simulation that would have taken a single desktop computer over 35 years to undertake, a 1 million atom MD simulation of the small, icosahedral plant virus STMV, standing for satellite tobacco mosaic virus, was run for 50 nanoseconds using NAMD [135]. In another study, this time comprising in excess of 2.64 million atoms, the ribosome has been studied using MD [134,136]. *Rhodobacter sphaeroides* photosynthetic units, known to form inner membrane invaginations comprising light-harvesting complexes, LH1 and LH2, and reaction centres, have been simulated: large models comprising 9 or 18 dimeric light harvesting 1 complexes and 144 or 101 light harvesting 2 complexes, representing a total of 3,879 or 4,464 bacteriochlorophylls, were evaluated [137]. Yet, perhaps the largest, and thus the most interesting, structure amongst these large scale simulations is that of the synaptic vesicle. It did not start with a single x-ray structure or one built using symmetry, but built the kind of complex, stochastic system of which the cell is constructed. Using specially derived measurements of protein and lipid composition, vesicle size, density, and mass, a full structural model was constructed and simulated using MD [138].

Simulations conducted on this scale will be vital in trying to understand and successfully characterize the structure of the catalogue of cellular pathways and networks emerging from proteomics, transcriptomics, and other high throughput approaches to functional genomics. Not simulations of precisely if inexactly defined – or should that be exactly but imprecisely defined – molecular structures, such as arise from X-ray or electron crystallography or multi-dimensional NMR. Rather, simulations of large-scale, semi-stochastic structures that define the ultra-structure of the cell. These simulations may focus on protein complexes or the behaviour of membranes, or of proteins concentrated within confined spaces, of supramolecular complexes, such as proteasome or inflammasome, or any of a hundred other things.

Many of these examples are of a size and complexity beyond the limits of current simulation technology. There are two ways to address the discrepancy between our aspirations and what is pragmatically close-at-hand: one is coarse-graining and the other is the deployment of petascale computing. Petascale is the new high-frontier of supercomputing. Recently, Saksena *et al*. [139] have reported the application of so-called petascale computing resources to computational science research in order to achieve results on an unprecedented scale, at an unprecedented resolution, and at an unprecedented speed. Utilising the then largest supercomputer in the world open to scientific research, they reported a number of distinct applications, including several in biomedicine, such as HIV and cerebrovascular haemodynamics. By exploiting exceptional scaling performance on up to 32768 cores, they showed that parallel programming with MPI is likely to be practical and useful when undertaken at the petascale in the near to medium future. More recently, they were able to extend this work to simulations running on over 65636 cores running via the IBM Blue Gene/P system. However, despite such prodigious feats, access to such systems remains highly restricted and thus simultaneously utilising other approaches remains a pragmatic strategy.

However, to have any chance of modelling some of the larger structures we list we must make recourse to the restrictions that very large scales imply, necessitating the use of both coarse-graining and simulation on a hierarchy of length and time scales. A wider spectrum of length and time scales can be reached by coarse-grained descriptions of large-scale phenomena [140]. MD simulation, with coarse-grained descriptions, is the only currently available method that can provide spatio-temporal information on large cellular systems [86,141].

Such a mesoscopic model would model each protein or each protein domain as a soft ellipsoidal particle. In such a model, the number of particles is dramatically reduced and a microsecond time step replaces the femtosecond one characteristic of MD. This will make tractable much larger and longer simulations. Other approaches make use of hybrid techniques that model the dynamics of macromolecules in solution by coupling equations which account for fluctuating hydrodynamics with MD describing the macromolecule.

**Figure 4:**
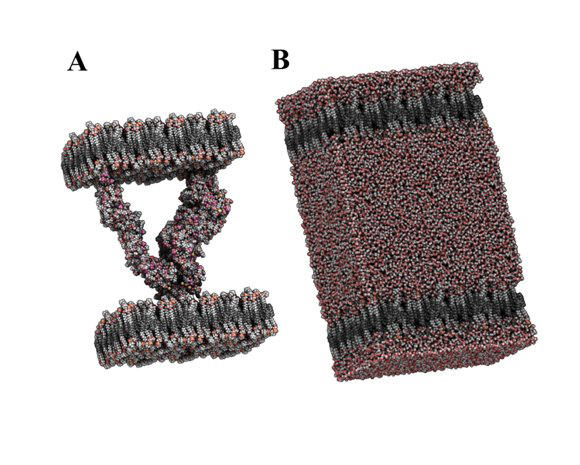
**Simulating the Immune synapse.** (A) A detailed molecular model used for the initial simulation of the immune synapse comprising CD4, peptide-MHC, TCR, and membrane regions [141]. (B) The same model as in (A), but this time it is shown fully solvated.

Water and lipid molecules are represented implicitly. Langevin dynamics, in which two additional force terms have been added to Newton's second law, account for omitted degrees of freedom [142]. One term represents a friction force which is proportional to the particle's velocity and is oppositely directed; the other is a stochastic term associated with the thermal motions of solvent and lipid molecules. A potential energy function is built which relates structure to energy and force, based around the use of the Gay-Berne potential [143], a generalized Lennard-Jones potential for ellipsoid particles. However, potential functions are useless until their parameters are optimized to represent real systems, here extant work is often used on the inter-molecular interactions of macrmolecules [87], to derive the parameter optimisation. Such simulations not only provide the pair-wise interaction forms in the effective potential, but also parse the interplay between the components involved in the immune response. The association and dissociation rate constants from experiments [90] would verify parameters obtained from simulation. See Figure 4.

Our coarse-grained particles move on the surfaces of lipid bilayers, a quasi two-dimensional environment. Lipid bilayers and solvent waters are modelled implicitly as a continuum to provide an effective potential, a friction drag, and a heat bath. The collective dynamics and elasticity of membranes affects the translation and localisation of proteins. The mature immune synapse, which is characterized by a stable distribution of membrane proteins, consists of two functional domains: a central cluster of TCR-pMHC complexes and a surrounding ring of adhesion molecules. Two segregated patterns in the junction are favourable because of the reduced energetic cost of bending the cell membranes to accommodate two pairs of rings separately. The differences in length between these regions create a functionally important supramolecular structure at the mesoscale and beyond. Inspection of such models allows effective comparison simulation with experiment, though in this case experiment results would be provided by real-time confocal microscopy rather than the time and space averages offered by x-ray crystallography. An effective potential term [144] represents the global deformations of the two cells at the junction. A good membrane potential including the bending elastic energy of lipid membrane is critical to mimicking the spontaneous curvature of the bilayer.

In summary, we can say with confidence that, eventually if not quite tomorrow, as more sophisticated methods evolve and develop, and MD becomes a widely and habitually used tool, science will, in concert with richer and improved measured data, develop ever more accurate and predictive simulations of biological relevant systems modelled at length and time scales appropriate to the problem. MD will ultimately break free from the many restrictions imposed by the limited data we currently possess by affording us the opportunity of true *de novo* prediction of equilibrium binding kinetic constants. Beyond that we envisage running simulations that pose biological questions that can only be answered by experiment, which in turn will drive the design of experiments. The immunological synapse has been suggested to be a therapeutic target [145]. The information provided by these research initiatives gives unprecedented insight into the details of immune synapse formation and structure that should, in turn, facilitate development of new approaches to the design of vaccines and immunomodulatory drugs.

## Conclusions

MD simulation [146] can be seen as a "virtual experiment" where atoms and molecules interact under the control of physical laws. The time evolution of atoms within the system is followed by integrating their Newtonian equations of motion. MD studies in biology have proliferated rapidly in recent years [134,146] as an important complement to experiment. These studies enable science to learn something new, something that cannot be found in other ways. MD simulations provide the ultimate details of molecular phenomena and enhance our understanding of biological function. It enables us to measure effectively that which hitherto was not measurable.

In terms of modelling the association of MHC molecule with antigenic peptides, it is increasingly clear that the whole peptide contributes to affinity, and thus to T cell-mediated immunogenicity; effective models of binding must represent this adequately. This MD does implicitly, and our group has extensive and continuing experience of developing reliable tools for this kind of study. Using MD, we can both predict, and cyclically manipulate, the affinity and thus the immunogenicity of antigenic epitope peptides, in an allele-dependent manner.

Many kinds of experiment have indicated the duration of IS formation and the shape of the mature IS. Structural studies of molecules comprising the IS reveal atomistically resolved yet static views of the individual molecular recognition events involved. Reaction-diffusion equations have been used to describe the spatiotemporal evolution of intracellular protein concentrations. Multi-scale MD simulations may be the best opportunity we have to reach a full understanding of this remarkable supra-macromolecular event at a cell-cell junction. The scale of simulations has now reached the point where these studies become possible. This is due to the advent of high-end parallel platforms like HECToR and grid infrastructure which achieves higher throughput computing by taking advantage of many networked computers.

Other than MD, many other approaches exist to model the interaction between TCR, peptide, and pMHC or between peptide and MHC. Various tools for both sequence-based approaches and structure-based T-cell epitope prediction are available [6,8,147,148]. However, there is still much space for improving T-cell epitope predictions [23,149], especially structural T-cell activation. Although MD approaches are increasingly seen as successful, even by their former critics, many such techniques retain the enduring limitations that have dogged atomistic simulation of macromolecules since its inception. Nonetheless, such techniques present us with an enticing avenue to access detail not known or knowable from other approaches. There is and will be a need to progress constantly to larger and larger simulations and to use the technique to drive rather than follow experiment.

While both MD and related methods hold out the greatest hope for true de-novo predictions of MHC binding, their present success rate is very much lower than that of data driven models. However, as with most of science, one must tease the genuinely useful from the self-aggrandizing hyperbole. Much work remains to be done on developing, refining, and applying this methodology.

## Abbreviations

AI: artificial intelligence; APCs: antigen-presenting cells; CG: coarse graining; CLIP: invariant chain-associated peptide; CPU: central processing unit; FEP: free-energy-perturbation; GPU: Games processing unit; IS: immune synapse; ITC: isothermal titration calorimetry; MC: monte carlo; MD: Molecular Dynamics; MHC: major histocompatibility complex; MLR: multiple linear regression; MM/GBSA: molecular mechanics/generalized Born surface area; NMA: normal mode analysis; pMHC: peptide-major histocompatibility complex; PLS: partial least squares; QM/MM: quantum mechanics/molecular mechanics; SMD: steered molecular dynamics; STMV: satellite tobacco mosaic virus; SPR: surface plasmon resonance; TI: thermodynamic integration; TCR: T-cell receptor; VS: virtual screening.

## Competing interests

The authors declare no competing financial interests.

## Authors' contributions

DF wrote the paper. IM wrote the paper. SW wrote the paper. PC wrote the paper. MD wrote the paper. KP wrote the paper.
